# P02.19. Resilience training for depressed healthcare workers: results from 2 and 12 month followup

**DOI:** 10.1186/1472-6882-12-S1-P75

**Published:** 2012-06-12

**Authors:** J Dusek, H Emmons, C Denton, S Masemer

**Affiliations:** 1 Penny George Inst. for Health & Healing, Abbot Northwestern Hospital, Allina Health, Minneapolis, USA

## Purpose

To evaluate the impact of the Resilience Training (RT) Program on depressive symptomology, QoL and presenteeism at 2 and 12 month follow-up assessments.

## Methods

Forty healthcare workers with major depressive disorder were enrolled in the study. The first 20 eligible employees who responded to study advertisements were assigned to RT program. The second 20 eligible employees had an 8 week waiting period before starting the RT program. Primary outcomes were changes in depression, QoL, and presenteeism. For the RT group, questionnaires were completed before and after the 8-week RT program. The wait-list group completed questionnaires before and after an 8-week wait period and after completion of the RT. Both groups also completed the questionnaires 2 and 12 months after their completion of the RT program.

## Results

Overall, 34 of the 40 participants completed the 2-month follow-up. Averaging results across all 34 participants, PHQ-9 scores dropped from 11.4 at initial baseline to 4.3 at 2 months (p<.0001) with 74% achieving remission (PHQ-9 of less than 5). The SF-12: mental status subscale improved from 32.4 to 47.5 (p<.0001). Presenteeism decreased from 34.2% to 10.9% (p<.0001). Using established procedures, this reduction in presenteeism translates to a cost savings of over $2,169 per participant over the 2-month period. The results persisted through the 12 month assessment. Averaging results across all 24 participants who completed the 12 month follow-up, PHQ-9 scores remained low with a score of 4.6 and 67% remained in remission. SF-12 scores remained high at 46.6. Presenteeism scores remained low at 7.9% and with cost savings of $13,037 per participant over the 1 year time period.

## Conclusion

Results from both 2 and 12 month follow-up assessments demonstrate that the RT program significantly improves clinical outcomes (depression and QoL) and could provide a significant return on investment if used in a healthcare workplace setting.

